# Evaluation of the Impact of Intratumoral Heterogeneity of Esophageal Cancer on Pathological Diagnosis and P16 Methylation and the Representativity of Endoscopic Biopsy

**DOI:** 10.3389/fonc.2021.683876

**Published:** 2021-08-17

**Authors:** Yu Qin, Jing Zhou, Zhiyuan Fan, Jianhua Gu, Xinqing Li, Dongmei Lin, Dajun Deng, Wenqiang Wei

**Affiliations:** ^1^National Cancer Registry Office, National Cancer Center/National Clinical Research Center for Cancer/Cancer Hospital, Chinese Academy of Medical Sciences and Peking Union Medical College, Beijing, China; ^2^Key Laboratory of Carcinogenesis and Translational Research, Peking University Cancer Hospital, Beijing, China; ^3^Department of Pathology, Peking University Cancer Hospital, Beijing, China

**Keywords:** esophageal cancer, P16 methylation, intratumoral heterogeneity, endoscopic biopsy, representativity

## Abstract

**Background:**

P16 methylation is expected to be potential diagnostic and therapeutic targets for esophageal cancer (EC). The intratumoral heterogeneity (ITH) of EC has been mentioned but has not been quantitatively measured yet. We aimed to clarify the impact of ITH on pathological diagnosis and P16 methylation, and the concordance between endoscopic biopsy and the corresponding surgically resected tissue.

**Methods:**

We designed a systematic sampling method (SSM) compared with a general sampling method (GSM) to obtain EC tumor tissue, tumor biopsy, and normal squamous epithelium biopsy. MethyLight assay was utilized to test P16 methylation. All specimens obtained by the SSM were pathologically diagnosed.

**Results:**

A total of 81 cases were collected by the GSM, and 91.4% and 8.6% of them were esophageal squamous cell carcinomas (ESCCs) and esophageal adenocarcinomas (EADs), respectively. Nine SSM cases were 100.0% ESCCs. The positive rates of P16 methylation of the GSM tumor and normal tissues were 63.0% (51/81) and 32.1% (26/81), respectively. For SSM samples, tumor tissues were 100.0% (40/40) EC and 85.0% (34/40) P16 methylated; tumor biopsy was 64.4% (29/45) diagnosed of EC and 68.9% P16 methylated; the corresponding normal biopsies were 15.7% (8/51) dysplasia and 54.9% (28/51) P16 methylated. The concordance of pathological diagnosis and P16 methylation between tumor biopsy and the corresponding tumor tissue was 75.0% and 62.5%, respectively.

**Conclusion:**

The SSM we designed was efficient in measuring the ITH of EC. We found inadequate concordance between tumor biopsy and tissue in pathological diagnosis and P16 methylation.

## Introduction

Esophageal cancer (EC) is a fatal upper gastrointestinal malignant tumor. Approximately, there were 604,100 new EC cases and 544,076 deaths worldwide in 2020, ranking 7th and 6th in all cancer, respectively (1). The concealment of EC occurrence led to a poor clinical outcome of EC that associated with late diagnosis at advanced stage and therapy resistance, resulting in a poor 5-year survival rate of 30.3% (2). It is crucial to facilitate the EC precise diagnosis and therapy.

Recently, studies have characterized that the CpG island of the *P16/CDKN2A* promoter is highly methylated in EC and might be a promising biomarker for EC personalized treatment and prognosis prediction (3–5). Since only a single sample was obtained to represent the methylation status of the whole tumor in present studies generally (6, 7), a wide variation of *P16* methylated rate was noticed in different studies that based on EC tissues, 40.0%–90.0%, which might be caused by ITH (intratumoral heterogeneity) that hindered its clinical application (8–14). Many studies uncovered the adverse effects of ITH on the cancer precise molecular classification, biomarker screening, and individualized precise treatment (15–17). Researches revealed that ITH can significantly affect the DNA methylation status of malignant tumors including breast cancer and lymphoma (15, 18). Since few studies focused on the impact of ITH on *P16* methylation in EC (6, 19), how much it impacts on *P16* methylation is still unclear.

In this study, we measured the *P16* methylation level in EC and evaluated the impact of ITH on *P16* methylation quantitatively to provide novel insights into the relationship between *P16* methylation and EC.

## Materials and Methods

### Subject Enrollment

In December 2019, EC inpatients who received esophagectomy and qualified the enrollment criteria were recruited from Linzhou Cancer Hospital, Henan province, a high incidence area of EC in China. Information including demographic characteristics, risk factors, and clinical diagnosis was collected.

#### Enrollment Criteria

1. Have not received radiotherapy or chemotherapy before operation.
2. *In vitro* time of surgical left tissue is less than an hour.
3. For the systematic sampling method, the length from the incisal edge to the tumor boundary that on the same side should be 3 cm at least.

### Human Research Subjects’ Protection

Written informed consent was obtained from all the participants. Ethics approval was provided by the Institutional Review Board of the Cancer Hospital of Chinese Academy of Medical sciences (No.15-151/1078).

### Sampling Method

(1) General sampling method (GSM)

Obtain one piece of tissue at the tumor region randomly and one piece from the surgical margin using scalpel, respectively.

**Figure 1:**
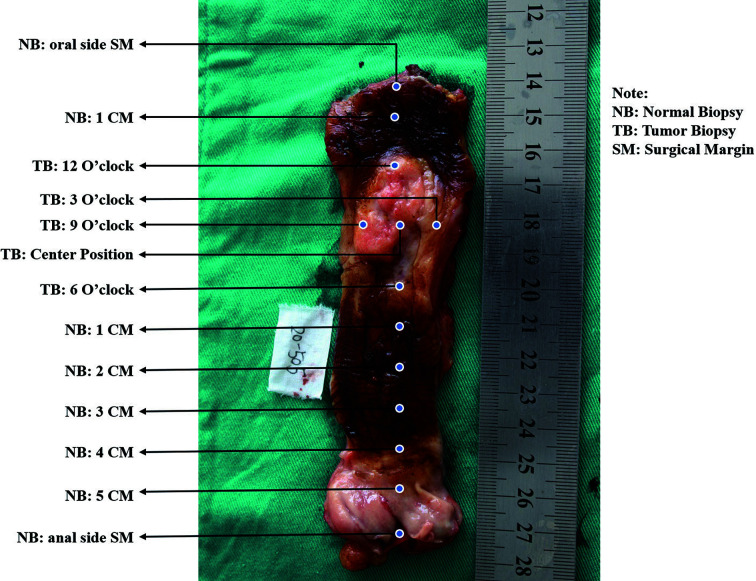
Diagrammatic layout of systematic sampling method.

(2) Systematic sampling method (SSM)

- See the esophagus tumor shape as a clock, and take mucosal biopsies at 3, 6, 9, and 12 o’clock and the center point using endoscopic biopsy forceps, respectively (**Figure 1**).
- Obtain the paired tumor tissue sample right under the biopsied site using the forceps-biting marks as guides by a scalpel.
- Biopsy from the normal surgical margin of tumor every centimeter.

All samples were store at -80°C.

### Pathological Diagnosis

All samples were shipped to the laboratory at Peking University Cancer Hospital and Institute. Tissues obtained by the SSM were fixed with formalin solution and paraffin embedding and hematein eosin (H.E.) staining by experienced technologists. Histological slides were reviewed and diagnosed by senior pathologists from the Department of Pathology of Beijing Cancer Hospital.

### Genomic DNA Preparation and Bisulfite Conversion

The frozen tissues obtained by the GSM were defrosted and grinded for genomic DNA extraction. For SSM samples, 8 to 10 pieces of 5-μm paraffin-embedded tissue were cut for manual DNA extraction, and 50 μl genomic DNA was eluted by Tris and EDTA (TE) buffer eventually.

Genomic DNA was modified by a DNA gold methylation kit (Zymo, Irvine, USA). Genomic DNA samples extracted from MGC803 and RKO cell lines were used as the *P16* methylation negative and positive control, respectively.

### Real-Time qPCR Procedure

We designed a useful and practical 115-bp MethyLight assay, reported previously, with very high specificity for the detection of *P16* methylation clinically (20). The *COL2A1* gene was selected as the internal reference gene. Gene sequences are listed below.

Methylated *P16* primer set: upstream 5′-cgcggtcgtggttagttagt-3′ and downstream 5′-tacgctcgacgactacgaaa-3′; methylated *P16* probe: 5′-6FAM-gttgtttttcgtcgtcggtt-TAMRA-3′; *COL2A1* primer set: upstream 5′-tctaacaattataaactccaaccaccaa-3′ and downstream 5′-gggaagatgggatagaagggaatat-3′; *COL2A1* probe: 5′-6FAM-ccttcattctaacccaatacctatcccacctctaaa-BHQ-1-3′ Annealing temperature is 58.5°C.

### Quality Control of qPCR Panel

We set three replications for each sample. The following were the criteria for judging experimental results: the cycle threshold (CT) value is valid only if ≥2 wells have a CT value, and the value is less than 40 for both. According to our previous study, we set 29.3 as the cutoff CT value of the internal reference gene *COL2A1* for the MethyLight assay result to reduce false negative (21). The *COL2A1* gene CT value for MGC803 cells in a different panel was set equal to the first panel to eliminate the systematic bias.

### Statistical Analysis

Demographic information such as age, gender, risk behaviors, and clinical characteristic was quantified. The positivity rates of *P16* methylation between different subgroups were compared by using McNemar chi-square test. Paired Student’s t test was used to compare the difference of ΔCt values for *P16* methylation between the tumor tissue and surgical margin tissues obtained by the GSM. Trend test was used in comparing the positive rates of *P16* methylation between tumor tissues with different grade dysplasia. *P* values less than 0.05 (two-sided) were statistically significant. Statistic Package for Social Science (SPSS) version 19.0 was used to analyze the data.

## Results

### General Characteristics of Participants and Sample Diagnosis

Ninety EC patients were recruited, and their characteristics are shown in **Table 1**. Among the 81 cases collected by the GSM, 74 (91.4%) were esophageal squamous cell carcinomas (ESCCs) and 7 (8.6%) were esophageal adenocarcinomas (EADs), and all 9 cases obtained by the SSM were ESCCs. One SSM case failed to be obtained tumor tissue due to the thinness of tumor. In total, 81 EC tumor tissues and the corresponding surgical margin tissues were collected by the GSM; 45 corresponding tumor biopsies (CTBs), 40 corresponding tumor tissues, and 51 corresponding surgical margin normal biopsies (CNBs) were collected by the SSM.

**Table 1:** General characteristics of participants.

Characteristics	N, (%)
Age (Mean ± SD)	64.8 ± 7.7
Gender	
Male	71 (78.9)
Female	19 (21.1)
Cigarette	
Yes	54 (60)
No	36 (40)
Alcohol	
Yes	48 (53.3)
No	42 (46.7)
Family history	
Yes	62 (68.9)
No	28 (31.1)
Pathological subtype	
ESCC	74 (91.4)
EAC	7 (8.6)
T stage	
1b	9 (10.0)
2	16 (17.8)
3	61 (67.8)
4a	4 (4.4)
N stage	
0	39 (43.3)
1	31 (34.4)
2	13 (14.4)
3	7 (7.8)
M stage	
0	88 (97.8)
1	2 (2.2)

ESCC, represents esophageal squamous cell carcinoma; EAC, represents esophageal adenocarcinoma.

### *P16* Methylation in GSM Samples

Generally, 63.0% (51/81) tumor tissues and 32.1% (26/81) paired normal tissues were *P16* methylated; 75.6% tumor tissues from habitual drinkers were *P16* methylated, which was significantly higher than those from nondrinkers (*p*<0.05). For normal tissues, males had a higher *P16* methylation positive rate than females (38.1% verse 11.1%; *p*<0.05). Patients at the advanced stage had a higher *P16* methylation positive rate in both tumor and normal tissues than those at the early stage, though statistically insignificant (**Table 2**).

**Table 2:** P16 methylation in GSM samples.

	Tumor [N, (%)]	*p*	Normal [N, (%)]	*p*
Overall (n=81)	51 (63.0)		26 (32.1)	0.001
Gender				
Male (n=63)	39 (61.9)	0.71	24 (38.1)	0.03^*^
Female (n=18)	12 (66.7)		2 (11.1)	
Age				
≤59 (n=16)	8 (50.0)	0.25	3 (18.8)	0.43
60-69 (n=44)	27 (61.4)		16 (36.4)	
≥70 (n=21)	16 (76.2)		7 (33.3)	
Smoke				
Yes (n=51)	35 (68.6)	0.17	19 (37.3)	0.20
No (n=30)	16 (53.3)		7 (23.3)	
Alcohol				
Yes (n=41)	31 (75.6)	0.02^*^	16 (39.0)	0.18
No (n=40)	20 (50.0)		10 (25.0)	
Family history				
Yes (n=57)	39 (68.4)	0.12	42 (33.3)	0.71
No (n=24)	12 (50.0)		12 (29.2)	
Pathological subtype				
ESCC	46 (62.3)	0.94	23 (31.8)	0.83
EAC	5 (71.4)		3 (42.9)	
Tumor stage				
T_1-2_ (n=23)	13 (56.5)	0.45	7 (30.4)	0.84
T_3-4_ (n=58)	38 (65.5)		19 (32.8)	
N_0_ (n=36)	21 (58.3)	0.44	10 (27.8)	0.46
N_1-3_ (n=45)	30 (66.7)		16 (35.6)	

*the difference is significant.

### Pathological Diagnosis of SSM Samples

In the SSM sample set, 40 tumor samples were taken from 8 patients by typical pathologic sampling from surgical resected tumor mass/lesion under endoscope-biting sites. All these 40 samples were diagnosed as ESCC and thus used as the golden standard. However, for the 45 CTB biopsies taken from the same tumor set, only 29 (64.4%) were diagnosed as ESCC, 10 (22.2%) as high grade dysplasia (HGD), 5 (11.1%) as moderate grade dysplasia (MGD), and 1 (2.2%) as mild grade dysplasia (mGD), respectively, indicating an unavoidable false negative ESCC detection by individual endoscopic examinations/biopsies alone. In contrast, 51 CNB biopsies from the corresponding surgical margins were diagnosed as follows: 0 ESCC, 2 HGDs, 2 MGDs, 4 mGDs, and 43 (84.3%) normal epitheliums, indicating a very high specificity to detect ESCC by endoscopic examination (**Table 3**).

**Table 3:** The results of pathological diagnosis of all 136 SSM samples taken from 40 surgical ESCC mass samples.

Sample type	Pathological diagnosis (%)					Total
	Normal	mGD	MGD	HGD	ESCC	
Tumor tissue mass	0	0	0	0	40	40 (100.0)
CTB	0	1 (2.2)	5 (11.1)	10 (22.2)	29 (64.4)	45 (100.0)
CNB	43 (84.3)	4 (7.8)	2 (3.9)	2 (3.9)	0	51 (100.0)

CTB, corresponding tumor biopsy; CNB, corresponding normal biopsy.

### *P16* Methylation in SSM Samples

In total, 68.9% (31/45) tumor biopsies were *P16* methylation positive, and no significant difference was found when comparing to the tumor tissues obtained by the GSM (63.0%).

Around 58.3% (21/36) and 88.9% (8/9) tumor biopsies from the noncenter and center positions, respectively, were ESCC. For the noncenter position tumor biopsies, 69.4% (25/36) were *P16* methylated, and for the center position biopsies, 66.7% (6/9) were *P16* methylated (*p*>0.1).

The *P16* methylation positive rate for tumor biopsies diagnosed of ESCC was 72.4% (21/29), 70.0% (7/10) for HGDs, and 50.0% (3/6) for MGDs and mGDs, (*p*_trend_<0.05).

For all the above 40 ESCC tissues from 8 patients, 85.0% (34/40) were *P16* methylation positive while a 100.0% positive rate of *P16* methylation was noticed in tumor tissues from the center position, which was higher than that in the tumor tissues obtained by the GSM (63.0%; *p*<0.05).

Between tumor biopsy and corresponding tumor tissue samples, 75.0% (6/8) and 62.5% (5/8) patients showed inconsistent results of pathological diagnosis (Nos. 1, 3, 5, 7, 8, and 9) and *P16* methylation (Nos. 3, 4, 5, 8, and 9) (**Table 4**).

**Table 4:** Diagnosis and *p16* methylation status of 40 tumor tissues and 45 tumor biopsies.

Patients No.	Sample type	Position (O’clock)									
		3		6		9		12		Center	
		Diag	P16m	Diag	P16m	Diag	P16m	Diag	P16m	Diag	P16m
1	TB	ESCC	+	ESCC	+	HGD	+	ESCC	+	ESCC	+
	TT	ESCC	+	ESCC	+	ESCC	+	ESCC	+	ESCC	+
2	TB	ESCC	+	ESCC	–	ESCC	+	ESCC	+	ESCC	+
	TT	ESCC	+	ESCC	–	ESCC	+	ESCC	+	ESCC	+
3	TB	ESCC	+	HGD	+	HGD	–	ESCC	–	ESCC	+
	TT	ESCC	+	ESCC	+	ESCC	+	ESCC	+	ESCC	+
4	TB	ESCC	+	ESCC	–	ESCC	–	ESCC	–	ESCC	+
	TT	ESCC	–	ESCC	–	ESCC	+	ESCC	–	ESCC	+
5	TB	ESCC	+	ESCC	+	HGD	+	HGD	+	HGD	–
	TT	ESCC	–	ESCC	+	ESCC	+	ESCC	+	ESCC	+
6	TB	ESCC	+	ESCC	+	MGD	–	MGD	+	EC	–
	TT	NA	NA	NA	NA	NA	NA	NA	NA	NA	NA
7	TB	MGD	+	ESCC	+	HGD	+	MGD	+	ESCC	+
	TT	ESCC	+	ESCC	+	ESCC	+	ESCC	+	ESCC	+
8	TB	mGD	+	MGD	–	MGD	–	ESCC	+	ESCC	–
	TT	ESCC	+	ESCC	+	ESCC	–	ESCC	+	ESCC	+
9	TB	HGD	–	ESCC	+	ESCC	–	ESCC	+	ESCC	+
	TT	ESCC	+	ESCC	+	ESCC	+	ESCC	+	ESCC	+

TB, represents tumor biopsy; TT, represents tumor tissue. +, represents p16 methylated; -, represents p16 un-methylated.

Among all the above surgical margin biopsies, 15.7% (8/51) were diagnosed of squamous epithelial dysplasia, including 2 HGDs, 2 MGDs, and 4 mGDs, and 6 dysplasia (2 HGDs, 2 MGDs, and 2 mGDs) located at 1 cm, 1 mGD at 2 cm, and 1 mGD at 3 cm from the tumor mass/lesion (**Table 5**). Totally, 54.9% (28/51) of these CNB biopsies were *P16* methylation positive, consisting of 17 (60.7%) from 1–2 cm, 9 (32.1%) from 3–4 cm, and 2 (7.1%) from 5–6 cm, respectively (*p*_trend_<0.05) (**Table 5**). In “1–5 cm” subgroups, 91.7%, 50.0%, 50.0%, 30.0%, and 25.0% biopsies were *P16* methylation positive, respectively, showing a decreasing trend with the increase in the distance from the biopsy site to the tumor mass/lesion (*p _trend_*<0.05).

**Table 5:** Pathological diagnosis and *p16* methylation of SM biopsies.

No.	Direction		The distance to the tumor border (CM)^a^					
			1	2	3	4	5	6
1	O	Diag	MGD	N	N	N	N	
		P16m	+	–	+	–	–	
2	O	Diag	HGD	N	mGD	N	N	N
		P16m	+	–	+	–	+	+
3	O	Diag	HGD	N	N	N		
		P16m	–	–	+	–		
	A	Diag	MGD	N	N	N		
		P16m	+	–	–	–		
4	O	Diag	N	N	N			
		P16m	+	+	+			
	A	Diag	N	N	N	N	N	
		P16m	+	+	–	–	–	
5	O	Diag	N	N	N			
		P16m	+	+	–			
6	O	Diag	N	mGD	N	N	N	
		P16m	+	+	–	+	–	
7	O	Diag	N	N	N	N		
		P16m	+	+	–	–		
	A	Diag	N	N	N	N		
		P16m	+	+	+	+		
8	O	Diag	mGD	N	N	N		
		P16m	+	–	+	+		
9	O	Diag	mGD	N	N	N		
		P16m	+	–	–	–		

+, represents p16 methylated; -, represents p16 un-methylated. N, represents normal. ^a^The last sample of each row was obtained from the incisal edge. O, represents the oral edge and A, represents the anal edge.

## Discussion

Due to the unfavorable prognosis of advanced EC, a number of studies hoped to seek biomarkers that have a diagnostic value in the early stage of EC (19). *P16* methylation was found prevalent in EC tissues and demonstrated a promising diagnostic, therapeutic, and prognostic value (3, 5, 22–24). However, many studies have shown large variations in detecting *P16* methylation with a positive rate ranging from 45% to 88%, even in the same race and same sites (25–27). Researchers had already recognized the variation in EC and speculated that it might be influenced by ITH on tumor suppressor methylation (6, 7, 28).

In this study, we designed a systematic sampling method to measure the ITH quantitatively and the representativeness of tumor biopsy on pathological diagnosis and *P16* methylation. We found a positive rate of 63.0% on *P16* methylation in the tumor tissues that were obtained by the GSM, which is at the same level as others’ reports (27, 29). The positive rate of P16 methylation in nonposition-specific and center tumor tissue was 85.0% and 100.0%, respectively, which was significantly higher than that in the GSM samples. This suggests that the tumor tissue from the center position was the most representative of the whole tumor lesion compared to other positions. Thus, the ITH might influence the positive rate of *P16* methylation if only one piece of tumor tissue was collected randomly (19, 28, 30). Our current study recommends that tumor tissue should be obtained at the center of the EC tumor rather than other position in future studies. Other researchers also revealed it in EC and other malignant tumor including breast cancer, liver cancer, colorectal cancer, and Ewing sarcoma (15–18).

We also noticed *P16* methylation prevalent in the paired surgical margin obtained from EC patients, which was consistent with others’ studies (31, 32), revealing that *P16* methylation has already occurred and developed in the “normal” tissue, and the surgical margin is not the most appropriate blank control in EC biomarker screening (5).

The tumor biopsy specimen was commonly analyzed in a number of studies, especially in studies of EC screening and early diagnosis, to represent whole individual cases (33, 34). However, researchers found that one single biopsy did not represent tumor or lesions adequately. On the aspect of pathological diagnosis, our results revealed that more than a third (35.6%) of biopsies obtained from the EC tumor surface were diagnosed as non-EC. A significant pathological difference between noncenter and center biopsies was found (58.3% *vs.* 88.9%). There were two patients that could be pathologically diagnosed as EC by using four biopsies from different position at least, suggesting that inadequate diagnosis (false negative) could occur easily when pathological diagnosis was made based on only one biopsy. For *P16* methylation, a lower positive rate of 68.9% was found in tumor biopsies compared to tumor tissues (85.0%). Although a *P16* methylation positive rate between nonspecific position, clock position, and center position was insignificantly different, a positive correlation was found between the *P16* methylation rate and the severity of dysplasia lesions, which was consistent with other researchers’ reports (35). Since one-third of the biopsies were *P16* methylation negative even from the center position, we considered that the position of tumor biopsy scarcely influenced the detection of *P16* methylation. Nonetheless, a smaller number of biopsies might result in a higher false negative.

Among the 40 pairs of tumor biopsies and corresponding tissues, 25.0% were inconsistent on *P16* methylation results in total, and with a kappa value of 0.31, had shown a lowly concordant between the biopsy and surgically resected specimens from the same patients. Taking the *P16* methylation result for tumor tissues as the golden standard, a false negative rate of 23.5% and a false positive rate of 33.3% were noticed in tumor biopsies. However, Ken Hatogai et al. found that highly concordant between endoscopic biopsy and surgically resected specimens from the same EC patients in detecting PIK3CA mutation status with an overall concordance rate of 98.3% (178/181) (36), which is inconsistent with our results.

The esophageal squamous epithelium region between the tumor area and the surgical margin was considered as autologous blank control in many studies usually. However, some researchers discovered that the *P16* methylation might be positive even in a histologically normal sample of EC patients, with positive rates of 36.8% (32) and 38.3% (37), which is consistent with the rate of 33.3% in our study. A lower positive rate, ranging from 0.0% to 21.3%, was also found by other researchers (26, 38). Our results suggested that this controversial finding might be induced by the length of the esophageal squamous epithelium region and the normal tissue position. It is worth noting that the positive rate of *P16* methylation increased to approximately 60.0% suddenly if the biopsy position moved forward just 1 cm to the surgical margin, and it remains at a lower positive rate of *P16* methylation only when it reaches over 3 cm from the tumor lesion. This might result in an uncanny false positive rate and confuse other researchers. Therefore, when an autologous blank control is essential, a normal tissue should be obtained at a position 3 cm far from the tumor lesion if conditions permit.

There were several limitations in this study. Primarily, the sample size is small, and the EC patients were heterogenous, which reduced the reliability of our findings and caused a limited extend power. This is a pilot study on the representativity of pathological diagnosis and DNA methylation of tumor suppressors applied on EC screening. We will validate the results in a larger cohort in the future. Secondly, we tested *P16* methylation only, and the heterogeneity status of other tumor suppressors is still not clear yet.

## Conclusion

Significant impacts of ITH on pathological diagnosis and *P16* methylation of EC and the concordance between tumor tissue and biopsy samples were observed in our SSM analyses Several suggestions about proper sampling to improve the performance of endoscope examination/biopsy were recommended. More studies with a larger sample size, more biomarkers, and high-resolution methylation testing are needed to be implemented in a larger cohort to evaluate the sampling method in the future.

## Data Availability Statement

The raw data supporting the conclusions of this article will be made available by the authors, without undue reservation.

## Ethics Statement

The studies involving human participants were reviewed and approved by the Institutional Review Board of the Cancer Hospital of Chinese Academy of Medical Sciences. The patients/participants provided their written informed consent to participate in this study.

## Author Contributions

YQ, WW, and DD contributed to the conception and design of the study and wrote the manuscript. YQ, JZ, WW, and DD interpreted the results, literature search, and construction of tables and figures. DL reviewed the pathological slides. YQ, JG, and XL contributed to questionnaire information and specimen collection. All authors contributed to the article and approved the submitted version.

## Funding

This study is supported by the National Key R&D Program of China (2016YFC0901400, 2016YFC0901404) and the National Natural Science Foundation of China (81974493).

## Conflict of Interest

The authors declare that the research was conducted in the absence of any commercial or financial relationships that could be construed as a potential conflict of interest.

## Publisher’s Note

All claims expressed in this article are solely those of the authors and do not necessarily represent those of their affiliated organizations, or those of the publisher, the editors and the reviewers. Any product that may be evaluated in this article, or claim that may be made by its manufacturer, is not guaranteed or endorsed by the publisher.
